# PD1/PDL1 and TIM3/Gal9 expression in acute lymphoblastic leukemia: Gal-9 expression on leukemia stem cells as an independent prognostic parameter

**DOI:** 10.1186/s12885-025-14856-9

**Published:** 2025-09-12

**Authors:** Azza M. Kamel, Abdallah M. Almuslimani, Eman Z. Kandil, Marwa Hanafy, Mohammed AM Samra, Youssef MS Madney, Randa A. Osman

**Affiliations:** 1 Clinical Pathology Department, NCI, Cairo University, Cairo, Egypt; 2 Medical Oncology Department, NCI, Cairo University, Cairo, Egypt; 3 Pediatric Oncology Department, NCI, Cairo University, Cairo, Egypt; 4 National Cancer Institute, Fom El-Khalig Square, Kasr El-Aini St. , Cairo, 11796 Egypt

**Keywords:** Check point inhibitors, PD-1, PDL-1, TIM3, Gal-9, ALL

## Abstract

The introduction of immune checkpoint inhibitors (ICI) which are designed not to activate the immune system to attack tumors but to eliminate the inhibitory signals that block antitumor T-cell responses represents a breakthrough in the treatment of malignancies. However, studies in acute lymphoblastic leukemia (ALL) patients are scarce in number and in the parameters addressed. In the present study we investigated the expression of two ICI inhibitors, PD1 and TIM3 on BM T- lymphocytes and PDL1 and Gal9 on blast cells and leukemia stem cells (LSCs) in 85 newly diagnosed ALL patients using flow cytometry. The associations and correlations with different clinical and hematological parameters were investigated. The results revealed that higher expression of these markers is associated with poor prognostic parameters in some instances and good prognostic parameters in others. These findings indicate that different prognostic parameters may have different mechanisms of action. The results of Gal9 expression on LSCs are notable. Gal9 expression on LSCs was negatively correlated with the percentage of LSCs% (*r*=-0.414, *P* < 0.001) indicating that both the number and the biological characteristics of LSCs should be considered. Additionally, Low Gal9 expression on LSCs was an independent prognostic parameter for both OS (*p* = 0.016) and PFS **(***p* = 0.034). This finding has not been previously reported. In conclusion this work represents a comprehensive study of some ICIs in ALL. The expression of Gal9 on LCSs may be a potential candidate for targeted therapy.

**Supplementary Information:**

The online version contains supplementary material available at 10.1186/s12885-025-14856-9.

## Background

Acute lymphoblastic leukemia (ALL) is a malignant transformation and proliferation of lymphoid progenitor cells in the bone marrow, blood, and extramedullary sites [1]. B-precursor ALL (BCP-ALL) is the most common malignancy in childhood and adolescence [2]. Despite the marked progress in therapy, relapse is the most important cause of treatment failure [3]. This challenge requires the search for new prognostic parameters and the identification of potential therapeutic targets. The concept of immunotherapy was recently introduced, and a breakthrough was the introduction of immune check point inhibitors (ICIs) with the goal of not activating the immune system to attack tumors but rather removing the inhibitory signals that block antitumor T-cell responses; this concept was elegantly reviewed by Sharma and Allison [4] and Meng et al. [5].

Inhibitory immune checkpoint pathways have been widely studied as possible mechanisms of immune escape in cancer, and for some of them, particularly the cytotoxic T lymphocyte antigen 4 (CTLA-4), and the programmed cell death-1 (PD1) pathways, inhibitory antibodies (Abs) have garnered approval worldwide for the treatment of a broad spectrum of neoplastic diseases [6].

The expression of additional modulatory inhibitory immune checkpoint molecules including T-cell immunoglobulin and mucin domain 3 (TIM3), are emerging as important in dysfunctional lymphocytes [6]. The interaction of immune checkpoint blockade (ICB) on T lymphocytes with their ligands on leukemic cells, PD1 with PDL1 and TIM3 with lymphocyte activation gene-9 (Lag9), can lead to defective T-cell-mediated antitumor immunity and disease progression [7, 8].

Concurrently, frequent PD1 overexpression on tumor-infiltrating lymphocytes (TILs) increases the likelihood of successful treatment with checkpoint blockade [9].

The combined use of TIM3 blockade with PD1 blockade drugs could be more effective than blockade of either the TIM3 or PD1 alone [10].

Since leukemia stem cells (LSCs) were described in acute myeloid leukemia in 1997, extensive studies have been directed to identification and characterization of such cell populations in various tissues. To date, significant progress has been made in understanding LSCs, leading to the development of numerous LSCs-targeted therapies [11].

T-cell- tumor interactions are well studied in solid tumors [12]. In terms of hematological malignancies, AML has been well investigated as previously reviewed [13, 14], but studies in ALL patients are limited.

In this work, we aimed to investigate the expression of PD1/PDL1 and TIM3/Gal9 on BM T lymphocytes and leukemia cells (blasts and LSCs) in ALL patients, to investigate their associations and correlations with other clinical and hematological parameters and investigate their impact on response to therapy and survival.

## Materials and methods

### Patients

Our study included 85 newly diagnosed ALL patients. The sample included 47 males and 38 females with an age range of 0.7–75 years, a mean of 14.0 ± 17.6 years and a median of 6 years.

All patients presented to the pediatric and medical oncology departments of the National Cancer Institute (NCI), Cairo University, from March 2022 until October 2022. The study was performed in accordance with Helsinki guidelines for the protection of human subjects and approved by the Institutional Review Board (IRB) of the NCI, Cairo University (No. 201920069.3). Written informed consents were obtained from all participants or their guardians.

The study included all newly diagnosed ALL patients during the study period, children, and adults and both sexes. The exclusion criteria included associated malignancies and previous chemo or radiotherapy.

### Methods

All patients were subjected to a thorough medical history, clinical examination, chest X-ray and abdomen/pelvic sonography. Patients were diagnosed by standard methods including complete blood count (CBC), bone marrow (BM) examination, immunophenotyping (IPT), DNA index, cytogenetics and molecular genetics. All patients were evaluated for PD1 and TIM3 expression on T lymphocytes and PDL1 and Gal9 expression on blast cells and LSCs in the BM.

### Flow cytometric studies

The monoclonal antibodies (Mo Ab) used for ICI are presented in supplementary Table (1). Whole blood staining method was performed. For surface staining, 10 µl of each labelled Mo Ab was added to 100 µl of the BM sample, which was subsequently incubated in the dark for 20 min, hemolyzed with hemolysing solution containing NH4CL, washed, re-suspended in 500 µl PBS and then analyzed. For cytoplasmic staining, a mixture of 500 µl of 4% paraformaldehyde as a fixative, 500 µl of PBS and 5 µl Tween 20 as a detergent was added to the cells after Hemolysis and the mixture was incubated for 10 min. The cells were washed, 10 µl of each Mo Ab was added and the mixture was incubated for 30 min at 4 °C. The cells were washed, re-suspended in 500 µl of PBS and analyzed. Any antigen is considered positive when ≥ 20% of blast cells are stained above the negative control except for CD34, CD10 and MPO where ≥ 10% is considered positive [15].

A ten colors BD FACS Canto flow cytometer was used, about 100,000 events were acquired and analysis was performed with FacsDIVA software.

Minimal/Measurable residual disease (MRD) was determined based on the detection of leukemia-associated immunophenotypes (LAIPs) using a panel of monoclonal antibodies distributed across two 8–10 color tubes, each incorporating a consistent backbone of CD45, CD19, CD34, and CD10, along with a combination of additional markers including CD73, CD9, CD58, CD38, CD81, CD20, CD13, CD33, CD56, and CD2.

Analysis of PD1 and TIM3 expression on CD4 + and CD8 + T lymphocytes and PDL1 and Gal9 expression on blast cells and LSCs are presented in Supplementary Figures (1 & 2). Given that there are no reference values for ICI expression, the median was used to divide patients into high and low expression groups.

Children (1-≥18 years) were treated with St. Jude Total Study XV protocol [16], infants (< 1 year) were treated according to Interfant-99 protocol [17], and adults (> 18 years) according to German [18], or Dana Farber [19].

The response to therapy was evaluated by MRD on day 15 and day 42, as well as complete remission (CR) at the end of induction and survival. CR was defined by no blasts in the PB and < 5% in the BM, absence of extramedullary disease, absolute neutrophil count of ≥ 1.0 × 10^9^/L, platelet count of ≥ 100 × 10^9^/L and transfusion independence.

### Statistical analysis

Statistical analysis was performed using IBM© SPSS© Statistics version 22 (IBM© Corp., Armonk, NY, USA). Numerical data are expressed as means and standard deviations (SDs) or medians and ranges as appropriate. Qualitative data are expressed as frequencies and percentages. The Chi-square test or Fisher’s exact test are used to examine the relationship between qualitative variables. For nonnormally distributed quantitative data, comparisons between two groups are done using Mann-Whitney test (non-parametric t-test). Correlations between numerical variables are tested using Spearman-rho correlation. Survival analysis is performed using Kaplan-Meier method and comparison between two survival curves is performed using log-rank test. Multivariate analysis is performed using Cox-proportional hazard regression model for the significant factors affecting survival on univariate analysis. The hazard ratio (HR) with its 95% confidence interval (CI) was used for risk estimation. All tests were two-tailed. A p-value < 0.05 was considered significant.

## Results

The study included 85 newly diagnosed ALL patients including 47 males and 38 females with ages ranging from 0.7 to 75 years, a mean age of 14.0 ± 17.6 years and a median age of 6 years. Sixty-five patients were children aged 0.7–18 years, with a mean age of 5.8 ± 4.7 years and a median age of 4 years; 16 patients (24.6%) were < 1 or ≥ 10, and 49 (75.4%) were aged 1 to < 10 years. Twenty patients were adults with ages ranging from 20 to 75 years with a mean age of 41.0 ± 17.1 years and a median age of 39 years; 9/20 patients (45.0%) were < 35 years, whereas 11 (55.0%) patients were ≥ 35 years.

Hepatomegaly was encountered in 38/84 (45.2%) patients, splenomegaly in 39/84 patients (46.4%), and lymphadenopathy in 43/84 (51.2%) patients. There was no Cerebro-spinal fluid (CSF) infiltration in any patient. In children, Hepatomegaly was encountered in 28/64 (43.8%) patients, splenomegaly in 27/64 (42.2%) patients, and lymphadenopathy in 37/64 (57.8%) patients. In adults, Hepatomegaly was encountered in 10/20 (50.0%) patients, splenomegaly in 12/20 (60.0%) patients, and lymphadenopathy in 6/20 (30.0%) patients.

Hematological findings, and CD34 and lymphocyte subsets in pediatric patients and adults are presented in Table (1).

**Table 1:** Hematological findings and CD34 and lymphocyte subsets in 85 acute lymphoblastic leukemia patients

**Parameter**	**Whole group** (N=85)	**Pediatric** (N=65)	**Adults** (N=20)
Hb: gm/dl	8.1 ±1.9a 8.0 (3-13.9)	8.0±1.9 8.0 (3-12.0)	8.5±1.7 8.0 (6.6-13.9)
TLC x10^9^/L	60.8± 158.9 12.0 (0.6-996.2)	63.8±176.0 11.8 (2.5-996.2)	51.1±84.6 17.9 (0.6-344)
PLT x10^9^/L.	70.9 ±146.7 37.0 (4-1304)	77.8±164.0 42.0 (4 −1304)	48.2±61.9 30.0 (6-277)
PB Blasts %	50.5±30.8 50 (2-98)	48.3±29.9 42 (2-98)	57.4±33.3 64 (3-95)
BM Blasts %	85.5±13.7 90 (20-98)	86.3±13.0 90 (20-98)	83.1±15.7 90 (41-96)
BM lymphocytes%	6.047±6.708 5 (0-51)	5.6±4.5 5 (0-18)	7.7±11.3 5 (0-51)
CD34 +	40.7±34.0 35.7 (1.0-98.6)	38.4± 32.6 35.7 (1.3-97.1)	48.0±38.2 42.1 (1.0-98.6)
CD34+/CD38 -	35.6±32.5 26.9 (0.2-96.7)	33.8 ±31.7 23.1 (0.2-96.7)	41.6±35.3 31.6 (0.8-94.2)
CD4+/CD3+	16.7±10.1 15.2 (1.3-40.9)	17.7±10.3 16.4 (1.3-40.9)	13.4±8.8 9.9 (3.1-31.6)
CD8+/CD3+	17.3±9.8 16.7 (1.4-39.6)	17.3± 9.4 17.7 (2.3-39.6)	17.2±11.5 14.3 (1.4-36.6)
CD4:CD8 Ratio	1.1±0.5 1.0 (0.3-2.4)	1.1±0.5 1.0 (0.3-2.4)	1.0±0.5 0.8 (0.4-2.2)

*Hb* Hemoglobin, *TLC* Total Leukocyte count, *Plt* Platelets, *PB* Peripheral Blood, *BM* Bone Marrow

^a^ Mean ± SD, Median (range)

BM was hypercellular in 54/85 (63.5%) patients, normocellular in 19 (22.4%) patients and hypocellular in 12 (14.1%) patients. CALL was the predominant phenotype (53/85, 62.4%), followed by pre-B (20/85, 23.5%) and pro-B (11/85, 12.9%), and only one case (1.2%) had mature B phenotype.

### Molecular genetics and cytogenetic findings

t(1;19) was encountered in 5/46 (10.9%) patients, t(4;11) in 1/46 (2.2%) patients, t(12;21) in 3/44 (6.8%) patients, t(9;22) p190 in 2/46 (4.3%) patients and t(9;22) p210 in 4/46 (8.7%) patients. Cytogenetic risk stratification revealed 23 (46.9%) patients in the favorable-risk group, 19 (38.8%) patients in the intermediate-risk group and 7 (14.3%) patients in the high-risk group. Most patients were diploid with one hypodiploid and 3 hyperdiploid patients.

### Response to therapy

MRD was evaluated in 53 patients. On day 15, 16 (30.2%) patients achieved MRD < 0.1, 30 (56.6%) patients had MRD 0.1- <10 and 7 (13.2%) patients had MRD ≥ 10%. On day 42, 45/52 (86.5%) patients achieved MRD < 0.01 and 7 (13.2%) patients had MRD ≥ 0.01%.

At the end of induction therapy, 51/60 (85.0%) patients achieved CR, and 7/63 (11.1%) patients relapsed.

### Immune check point inhibitors (ICI) marker expression

ICI marker expression data are presented in Table (2). There was marked variability in the expression levels; the median value was taken to divide the patients into low expressor and high expressor groups.

**Table 2:** Immune check points inhibitors expression in 85 acute lymphoblastic leukemia patients

**Parameter**	**Mean ± SD.** **Median (Range)**	**Expression level**		**Parameter**	**Mean ± SD.** **Median (Range)**	**Expression level**	
		**Low: No (%)**	**High: No (%)**			**Low: No (%)**	**High: No (%)**
PD-1 on CD4 T lymphocytes	46.4 ± 22.5 49.3 (0.2-85.7)	42 (49.4)	43 (50.6)	PDL-1 on Blasts	12.3 ± 12.9 8.5 (0.6-59.9)	42 (49.4)	43 (50.6)
PD-1 on CD8 T lymphocytes	39.3 ± 20.9 39.6 (0.3-78.2)	42 (49.4)	43 (50.6)	Gal-9 on Blasts	12.5 ± 15.4 7.9 (0.3-93.5)	42 (49.4)	43 (50.6)
TIM-3 on CD4 T lymphocytes	1.6 ± 3.4 0.7 (0.1-24.6)	42 (49.4)	43 (50.6)	PDL-1/Gal-9 on Blasts	4.6 ± 6.4 2.2 (0.1-29.7)	42 (49.4)	43 (50.6)
TIM-3 on CD8 T lymphocytes	1.6 ± 3.2 0.7 (0.1-22.8)	41(48.2)	44 (51.8)	PDL-1 on CD34+/CD38- LSCs	20.3 ± 18.1 15.3 (0.5-81.6)	42 (49.4)	43 (50.6)
PD-1/TIM-3 on CD4 T lymphocytes	1.1 ± 2.6 0.4 (0.1-17.3)	35 (41.2)	50 (58.8)	Gal-9 on CD34+/CD38- LSCs	20.3 ± 20.1 14.3 (0.6-96.8)	42 (49.4)	43 (50.6)
PD-1/TIM-3 on CD8 T lymphocytes	1 ± 2.4 0.3 (0.1-20.2	33 (38.8	52 (61.2)	PDL-1/Gal-9 on CD34+/CD38- LSCs	11.09±13.74 5.7 (0.3-72.3)	42 (49.4)	43 (50.6)

### PD1 expression on T lymphocytes was associated with male sex, Hb level in children and CALL phenotype (Table 3)

No associations were detected between PD1 expression on CD4 + or CD8 + T-lymphocytes and age, hepatomegaly, splenomegaly, lymphadenopathy, or CSF infiltration. However, high expression on both CD4 + and CD8 + was associated with male sex (p value 0.023 for both).

**Table 3:** Significant associations of PD-1 and TIM-3 on T lymphocytes in 85 acute lymphoblastic leukemia patients

**T -cell subset**	**variable**	**No**	**High expressors**	**Low expressors**	*P* value
PD-1					
CD4+ & CD8+	Gender: Males Females	47 38	29 (61.7%) 14 (36.8%)	18 (38.3%) 24 (63.2%)	0.023
CD4+ in children	Hb: gm/dl <8 >8	32 33	21 (65.6%) 13 (39.4%)	11 (34.4%) 20 (60.6%)	0.034
CD8+	IPT: CALL Pre-B Pro-B	53 20 11	33 (62%) 7(35%) 3(27.3%)	20 (38%) 13 (65%) 8 (72.7%)	0.041
CD8+	Day 15 MRD: < 0.1 0.1-<10 ≥10	16 30 7	6 (37.5%) 22 (73.3%) 3 (42.9%)	10 (62.5%) 8 (26.7%) 4 (57.1%)	0.042
TIM-3					
CD8+	TLC x10^9^/L: <10 10-<50 >50	39 28 10	25 (64.1%) 15 (53.6%) 3 (30%)	14 (35.9%) 13 (46.4%) 7 (70%)	0.026
CD8+	PB Blast %	85	34% (2-95)	70% (3-98)	0.007
CD4+	BM Blast%	85	88% (20-98)	92% (65-98)	0.03
CD8+	BM Blast%	85	88% (20-98)	92% (40-98)	0.051
CD8+	Day 42 MRD < 0.01	45	25 (55.6%)	20 (44.4%)	0.006
CD8+	CR	51	29(56.9%)	22(43.1%)	0.011
PD-1/TIM-3 Co-expression					
CD8+	TLC x10^9^/L: <10 10-<50 >50	39 28 10	29 (74.4%) 18 (64.3%) 4 (40%)	10 (25.6%) 10 (35.7%) 6 (60%)	0.005
CD8+	IPT: CALL Pre-B Pro-B	53 20 11	35 (66.0%) 14 (70%) 2 (18.2%)	18 (34%) 6 (30%) 9 (83.8%)	0.016
CD8+	PB blast %	85	34 (2-95)	77 (3-98)	0.004
CD4+	BM blast %	85	89 (20-98)	93 (65-98)	0.04

No associations were detected between PD1 expression on CD4 or CD8 T-lymphocytes and TLC, platelets, BM cellularity, PB Blast %, BM blast %, or BM lymphocyte %.

High expression on CD4 T lymphocytes in children, but not in adults, was associated with Hb < 8, 21/32 (65.6%) vs. 13/33 (39.4%) for children with Hb ≥ 8 gm/dl (*p* = 0.034). No association was detected with expression on CD8 T lymphocytes in either children or adults.

High PD1 expression on CD8 T lymphocytes, but not CD4 T lymphocytes, was associated with the CALL phenotype 33/53 (62.3%), vs. 3/11 (27.3%) for Pro-B phenotype, and 7/20 (35%) for Pre-B phenotype (*p* = 0.041). The only mature B phenotype patient had low expression on both CD4 and CD8 T Lymphocytes.

No associations were detected between PD1 expression on CD4 or CD8 T-lymphocytes and CD34%, CD34+/CD38-%, CD3+/CD4+%, CD3+/CD8+%, the CD4:CD8 ratio, molecular genetics, cytogenetic risk groups or the DNA index.

### PD1 expression on T lymphocytes was associated with day 15 MRD

Day 15 MRD 0.1-<10 was associated with high PD1 expression on CD8 + T lymphocytes, 22/30 (73.3%) vs. 6/16 (37.5%) for MRD < 0.1, and 3/7 (42.9%) for MRD ≥ 10 (*p* = 0.042). The corresponding values for CD4 + T lymphocytes were 19/30 (63.3%), 7/16 (43.75%) and 1/7 (14.3%) respectively with borderline significance (*p* = 0.051).

No associations were observed with MRD on day 42 or the achievement of CR at the end of induction.

### PD1 expression on T lymphocytes had negative correlations with CD8 + T cells and CD4:CD8 ratio

No correlations were detected except for a negative correlation of the CD4:CD8 ratio with PD1 expression on CD4 + T-cells (*r*=−0.227, *p* = 0.037, Supplementary Fig. 3a) and on CD8 + T-cells (*r* = 0.233, *p* = 0.032 Supplementary Fig. 3b).

### TIM3 expression on T lymphocytes was associated with TLC and blast% (Table 3)

No association were detected between TIM-3 expression on CD4 or CD8 T-lymphocytes and either age, gender, hepatomegaly, splenomegaly, lymphadenopathy, CNS infiltration, Hb level, platelet count, IPT, BM cellularity, BM lymphocytes%, CD34%, CD34+/CD38-%, CD3+/CD4+, CD3+/CD8+, CD4:CD8 ratio, molecular genetics, cytogenetic risk groups or DNA index.

Among the 39 patients with a TLC < 10 × 10^9^/L, 25 (64.1%) had high expression vs. 15/28 (53.6%) in patients with TLC 10-<50 × 10^9^/L and 3/10 (30%) in patients with TLC > 50 × 10^9^/L (*p* = 0.026). No association was detected with TIM3 on CD4 + T cells.

A higher PB blast percentage was associated with low TIM3 expression on CD8 + but not CD4 + T cells (*p* = 0.007). A higher BM blast percentage was significantly associated with low TIM3 expression on CD4 + T cells (*p* = 0.03) and had a borderline association with TIM3 expression on CD8 + T cells (*p* = 0.051).

### TIM3 expression on CD8 + T lymphocytes was associated with day 42 MRD and CR (Table 3)

There was no statistically significant association between TIM3 expression on CD4 or CD8 + T-lymphocytes and MRD on day 15. On the other hand, a statistically significant association was found between TIM3 high expression on CD8 + but not CD4 + T-lymphocytes and MRD on day 42 < 0.01 (*P* = 0.006).

Complete remission was achieved in 51/60 patients at the end of induction. There was no statistically significant relationship with TIM3 expression on CD4 + T-lymphocytes. On the other hand, CR was associated with high TIM3 expression on CD8 + T lymphocytes (*p* = 0.011).

### TIM3 expression on T lymphocytes had negative correlation with TLC and blast%

TIM3 expression on CD4 + T-lymphocytes was negatively correlated with TLC (*r*=−0.249, *p* = 0.022, Supplementary Fig. 3c) and initial BM blasts percentage (*r*=−0.352, *P* = 0.001, Supplementary Fig. 3 d).

TIM3 expression on CD8+ T-lymphocytes was negatively correlated with TLC (*r*=−0.249, *P*=0.022, Supplementary Fig. 3e), initial PB blasts percentage (*r*=−0.352, *P*=0.001, Supplementary Fig. 3f) and the percentage of BM blasts (*r*=−0.352, *P*=0.001, Supplementary Fig. 3 g).

### PD1/TIM3 co-expression on CD4 + and CD8 + T-lymphocytes was associated with TLC, blast% and CALL (Table 3)

No associations were detected between PD-1/TIM-3 co-expression on T lymphocytes and age, sex, hepatomegaly, splenomegaly, lymphadenopathy, Hb level, platelet count, BM cellularity, BM lymphocytes, CD34+%, CD34+/CD38-%, CD3+/CD4+%, CD3+/CD8+%, the CD4/CD8 ratio, molecular genetics, cytogenetic risk groups or the DNA index.

A TLC < 10 × 10^9^/L was associated with high co-expression on CD8 + T-cells (29/39, 74.4% vs. 10/39, 25.6%); whereas a TLC > 100 × 10^9^/L was associated with low expression (7/8, 87.5% vs. 1/8, 12.5% (*p* = 0.005).

A positive association was detected between high co-expression on CD8 + but not CD4 + T-cells and CALL (35/52, 66.0%, *p* = 0.016). PB blast percentage was associated with low co-expression on CD8 + but not CD4 + T-cells (77, range 3–98 vs. 34, range 2–95%, (*P* = 0.004).

A high BM blast percentage was significantly associated with low co-expression on CD4 + but not CD8 + T cells (93, range 65–98 vs. 89, range 20–98%, *p* = 0.04).

There was no statistically significant association between PD1/TIM3 co-expression on either CD4 + or CD8 + T-lymphocytes and MRD on day 15, MRD on day 42, or CR achievement at the end of induction.

### PD1/TIM3 co-expression on T lymphocytes had negative correlation with TLC and blast%

PD1/TIM3 co-expression on CD4 + T-lymphocytes was negatively correlated with TLC (*r*=−0.268, *P* = 0.013, Supplementary Fig. 4a) and the percentage of BM blasts (*r*=−0.307, *P* = 0.005, Supplementary Fig. 4b).

PD1/TIM3 co-expression on CD8+ T-lymphocytes was negatively correlated with TLC (r=−0.414, P<0.001, Supplementary Fig. 4c) and the initial PB blast percentage (r=−0.387, P<0.001, Supplementary Fig. 4 d).

### PDL1 expression on blasts was associated with CD4+%, CD8+%, and CD34% (Table 4)

#### PDL1 expression on LSCs was associated with hepatomegaly and t(12;21) (Table 5)

There was no statistically significant association between PDL1 expression on blasts or LSCs and age, sex, lymphadenopathy, or splenomegaly. Hepatomegaly was significantly associated with high PDL1 expression on LSCs (24/38, 63.2% vs. 14/38, 36.8%, *P* = 0.028) but not on blasts.

**Table 4:** Significant associations of PDL-1 and Gal-9 on blast cells in 85 acute lymphoblastic leukemia patients

**Parameter**	**No**	**High expressors**	**Low expressors**	*P* value
PDL-1				
CD4+/CD3+%,	85	12 (1.3-38.7)^a^	18.3 (2.4-40.9)	0.014
CD8+/CD3+%	85	15.2 (2.3-36)	18.9 (1.4-39.6)	0.030
CD34%,	85	53.6 (1-98.6)	16.7 (1.3-97.3)	0.004
Day 15 MRD <0.1 0.1-<10%,	30	17 (56.7%)	13 (43.3%)	0.047
Gal-9				
Day 15 MRD <0.1 0.1-<10%	16	4 (25.0%) 21 (70.0%)	12 (75.0%) 9 (30.0%)	0.001

^a^ Median (range)

No associations were detected between PDL1 expression on blasts or LSCs and Hb level, platelet count, TLC, BM cellularity, PB blast%, BM blast%, BM lymphocytes%, CD34+, CD34+/CD38-, CD3+/CD4+, CD3+/CD8 + or CD4/CD8 ratio, t(1;19), t(4;11), t(9;22) p190, t(9;22) p210, genetic risk stratification or the DNA index.

**Table 5:** Significant associations of PDL-1 and Gal-9 on CD34+/CD38- leukemia stem cells in 85 acute lymphoblastic leukemia patients

**Parameter**	**No**	**High expressors**	**Low expressors**	*P *value
**PDL-1**				
Hepatomegaly	38	24 (63.2%)	14 (36.8%)	0.028
**Gal-9**				
Hepatomegaly	38	25 (65.8%)	13 (34.2%)	0.015
PB blast %	85	64 (3-98)^a^	40 (2-94)	0.020
BM blast %	85	88 (20-98)	92 (41-98)	0.030
BM lymph %	85	5 (0-21)	4 (0-18)	0.034
CD34%	85	51.4 (2.1-98.6)	13.8 (1-96.2)	0.002
CD34+/CD38-%	85	8.1 (0.2-94.5)	46.6 (0.3-96.7)	0.002
**PDL-1/Gal-9 co-expression**				
CD34%	85	17.1 (1-97.3)	51.4 (2.1-98.6)	0.003
CD34+/CD38-%	85	11.3 (0-2-94.5)	46.0 (0.3-96.7)	0.002

^a^Median (range)

All 3 t(12;21) positive cases had low PDL1 expression on LSCs (*P* = 0.049), while there was no association with expression on blasts.

A significantly greater median CD4+/CD3+%, (*P* = 0.014) and CD8+/CD3+% (*P* = 0.030) were found in patients with lower PDL1 expression on blasts. A significantly greater median CD34% was found in patients with higher PDL1 expression on blasts (*P* = 0.004). No similar associations were found with the CD34+/CD38-% or the CD4/CD8 ratio.

### High PDL1 expression on blasts was associated with day 15 MRD (Table 4)

High PDL1 expression on blasts was significantly associated with positive day 15 MRD 0.1-<10% (*P* = 0.047); no similar association was found for expression on LSCs.

There was no association between PDL-1 expression on either blasts or LSCs and MRD on day 42 or CR achievement at the end of induction.

### PDL1 expressions on blast cells had positive correlations with CD34+% and CD34+/CD38-% and negative correlation with CD4+% and CD8+%

#### PDL1 expressions on LSCs had negative correlation with CD34+% and CD34+/CD38-%

PDL1 expression on blasts was positively correlated with the CD34 + percentage (*r* = 0.300, *P* = 0.005, Supplementary Fig. 5a) and the CD34+/CD38- percentage (*r* = 0.301, *P* = 0.005, Supplementary Fig. 5b) and negatively correlated with the CD4+/CD3 + percentage (*r*=−0.295, *P* = 0.006, Supplementary Fig. 5c) and the CD8+/CD3 + percentage (*r*=−0.218, *P* = 0.045, Supplementary Fig. 5 d). PDL1 expression on LSCs was negatively correlated with CD34+% (*r*=−0.225, *p* = 0.039, Supplementary Fig. 5e), and CD34+/CD38-% (*r*=−0.245, *p* = 0.024, Supplementary Fig. 5f).

### Gal9 expression on blasts was associated with t(9;22) and day 15 MRD (Table 4)

#### Gal9 expression on LSCs was associated with hepatomegaly, blast%, CD34+%, CD34+/CD38-%, BM T lymphocytes (Table 5)

No associations were detected between Gal9 expression on blasts and age, gender, lymphadenopathy, hepatomegaly, splenomegaly CNS involvement, TLC, PLT count, or Hb level. There were no significant association between Gal9 expression on LSCs and age, sex, lymphadenopathy, splenomegaly, TLC, PLT count, or Hb level.

High Gal9 expression on LSCs was associated with hepatomegaly (25/38, 65.8% vs. 13/38, 34.2%, *p* = 0.015).

No significant associations were detected between Gal9 expression on blasts and PB blasts%, bone marrow cellularity, BM blasts%, or BM lymphocytes%, IPT, CD34+, CD34+/CD38-, CD3+/CD4+, CD3+/CD8+%, or the CD4/CD8 ratio. A significantly greater median initial PB blasts % was found in patients with low Gal9 expression on CD34+/CD38- LSCs (*P* = 0.020).

No significant association was detected between Gal9 expression on LSCs and bone marrow cellularity. Low Gal9 expression on LSCs was associated with increased BM blasts% (*P* = 0.030), increased CD34%, and increased CD34+/CD38-% (*P* = 0.002).

A significantly greater median initial BM lymphocytes percentage was found in patients with higher Gal9 expression on LSCs (*P* = 0.034). No similar associations were found with the percentage of CD4+/CD3 + or CD8+/CD3 + T-cells or the CD4/CD8 ratio.

There was no significant association between Gal9 expression on blasts or LSCs and t(1;19), t(4;11), t (12;21), or t (9;22) p190, genetic risk stratification or the DNA index. All 4 t(9;22) p210 positive patients had high Gal9 expression on blasts (*P* = 0.045); there was no association with expression on LSCs.

Low Gal9 expression on blast cells was associated with negative MRD < 0.1 on day 15 (12/16, 75%vs. 4/16, 25%) and high expression was associated with day 15 + ve MRD 0.1-<10% (21/30, 70.0% vs. 9/30, 30.0%); all 7 patients with MRD ≥ 10 were low expressors (*P* < 0.001).

No similar association was found for Gal-9 expression on either blasts or LSCs for MRD on day 42 or CR.

No correlations were detected between Gal-9 expression on blast cells or LSCs and any clinical or hematological parameters.

### PDL1/Gal9 co-expression on blasts was associated with t(9;22), (Table 4)

#### PDL1/Gal9 co-expression on LSCs was associated with CD34+% and CD34+/CD38-% (Table 5)

No associations were detected with age, gender, hepatomegaly, splenomegaly, lymphadenopathy, CNS involvement, Hb, TLC, platelets, PB and BM blasts percentages, BM cellularity, BM lymphocytes, IPT, CD4+/CD3+, CD8+/CD3+ % T-cells, the CD4/CD8 ratio, cytogenetic risk stratification, the DNA index, t(1;19), t(4;11), t(12;21), or t (9;22) p190. For t(9;22) p210, all 4 positive patients had high co-expression of PDL1/Gal9 on blasts (*P* = 0.029).

There was no association between PDL1/Gal9 co-expression on blasts and CD34% or CD34+/CD38-% LSCs. Whereas PDL1/Gal9 co-expression on CD34+/CD38- LSCs was negatively associated with CD34% (*p* = 0.003) and CD34+/CD38-% (*p* = 0.002).

### PDL1/Gal9 co-expression on blast cells correlated with CD34+%, CD34+/CD38-% and CD4+/CD3+%

#### PDL1/Gal9 co-expression on LSCs correlated with BM lymphocyte%, CD34+%, and CD34+/CD38-%

PDL1/Gal9 co-expression on blasts was positively correlated with the CD34 + percentage (*r* = 0.240, *P* = 0.027, Supplementary Fig. 6a) and the CD34+/CD38- percentage (*r* = 0.237, *P* = 0.029, Supplementary Fig, 6b) and negatively correlated with the CD4+/CD3 + percentage (*r*=−0.253, *P* = 0.019, Supplementary Fig. 6c).

PDL1/Gal9 co-expression on CD34+/CD38- LSCs was positively correlated with BM lymphocyte percentage (*r* = 0.292, *p* = 0.008, Supplementary Fig. 6 d), and negatively correlated with CD34% (*r*= −0.458, *p* = 0.001, Supplementary Fig. 6e) and CD34+/CD38-% (*r*=−0.0463, *p* = 0.001, Supplementary Fig. 6f).

A summary of the significant correlations of the studied parameters is presented in Table (6).

**Table 6:** Significant correlations between immune check points inhibitors (ICI) expression and various parameters in 85 acute lymphoblastic leukemia patients

**T-cell**	**Parameter**	**r**	**p**	**parameter**	**r**	**p**
**PDI on T lymphocytes**				**PDL-1 on blasts**		
CD4+	CD4:CD8	−0.227	0.037	CD34+ %	0.300	0.005
CD8+	CD4:CD8	−0.233	0.032	CD34+/CD38-	0.301	0.005
**TIM-3 on T lymphocytes**				CD4+/CD3+%	−0.295	0.006
CD4+	TLC	−0.249	0.022	CD8+/CD3+ %	−0.218	0.045
CD4+	BM blast%	−0.352	0.001	**PDL-1/Gal-9 on blasts**		
CD8+	TLC	−0.249	0.022	CD34+ %	0.240,	0.027)
CD8+	PB blast%	−0.377	<0.001	CD34+/CD38- %	0.237	0.029
CD8+	BM blasts %	−0.352	0.001	CD4+/CD3+ %	−0.253	0.019
**PD1/TIM-3 on T lymphocytes**				**PDL-1 on LSCs**		
CD4+	TLC	−0.268	0.013	CD34+ %	−0.225	0.039
CD4+	BM blasts %	−0.307	0.005	CD34+/CD38- %	−0.245	0.024
CD8+	TLC	−0.414	<0.001	**Gal- 9 on LSC**		
CD8+	PB blasts%	−0.387	<0.001	CD34+ %	−0.394	<0.001
				CD34+/CD38- %	0.414	<0.001
				**PDL-1/Gal-9 on LSCs**		
				BM lymphocyte%	0.292,	0.008
				CD34%	−0.458,	0.001
				CD34+/CD38-%	−0.0463	0.001

### Survival analysis

The survival analysis included 80 patients; 5 patients lost follow-up and were excluded from the analysis. The median follow-up period was 12.7 (0.1–22.8) months. The median OS was not reached; however, the cumulative OS was 74.3%, at 6 months, 70.3% at 18 months, and 70.3% at the end of the study. The cumulative PFS rate was 73.0% at 6 months, 62.7% at 18 months, and 62.7% at the end of the study.

### Low Gal9 on CD34+/CD38- LSCs was associated with better OS

**Fig. 1:**
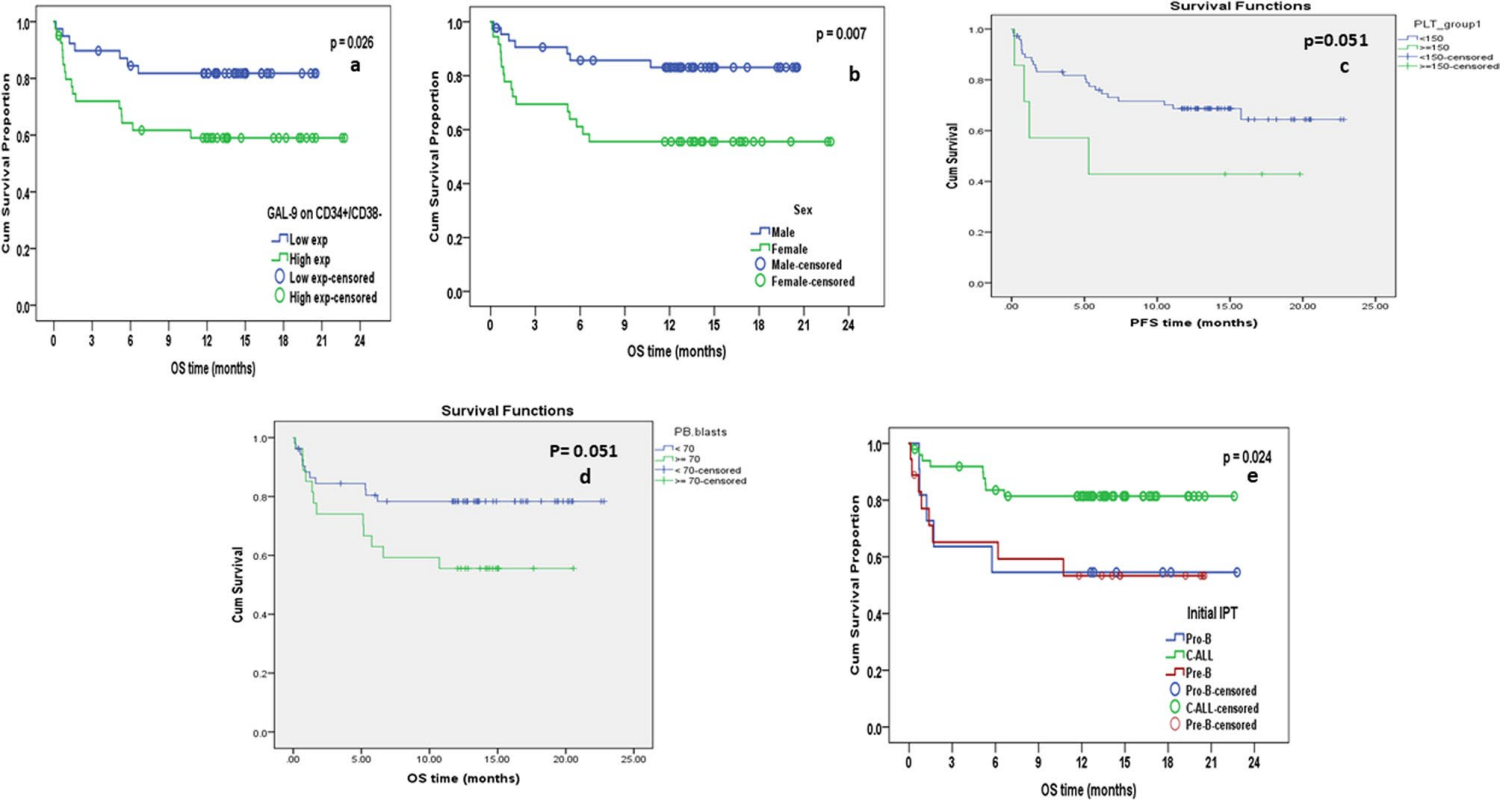
Factors that had significant Impact on overall survival in 80 acute lymphoblastic leukemia patients (using Kaplan-Meier method and log-rank test) (**a**) Gal9 expression on CD34+/CD38- LSCs, (**b**) Impact of sex (44 male and 36 female), C: platelet count (cutoff 150x10^9^/L), D: PB blast %, (cutoff 70%) (**e**) immunophenotype

There was no impact on OS of the expression of any ICP marker except for Gal9 on CD34+/CD38- LSCs; low Gal-9 expressors had significantly higher cumulative survival rates (81.7% vs. 59.0%, *p* = 0.026) (Table 7; Fig. 1a).

**Table 7:** Prognostic factors that significantly affected overall survival in 80 acute lymphoblastic leukemia patients

**Parameter**		**No.**	**No. of events**	**Cumulative survival at 18 M % **	***P*** value
Gal-9 on CD34+/CD38-	Low expressor	39	7	81.7	0.026
	High expressor	41	16	59.0	
Gender	Male	44	7	83.1	0.007
	Female	36	16	55.6	
PLT	<150	73	19	73.0	0.051
	≥150	7	4	42.9	
PB blasts %	< 70 ≥70	53 27	11 12	78.4 55.6	0.051
Initial IPT	Pro-B	11	5	54.5 a	0.024
	C-ALL	50	9	44 b	
	Pre-B	18	8	53.3 a

### Male sex, higher platelet count, lower blast% and Low Gal9 on CD34+/CD38- LSCs were independently associated with better OS

Prognostic factors that had an impact on OS are presented in Table (7) and Fig. (1). These included male sex (*p* = 0.007, Fig. 1b), platelet count ≥ 150 × 10^9^/L (*p* = 0.051, Fig. 1c), PB blasts < 70% (*p* = 0.051, Fig. 1 d), and the CALL phenotype (*p* = 0.024, Fig. 1e).

On multivariate analysis using Cox-proportional hazard model, the independent factors that significantly affected overall survival (OS) were sex (*p* = 0.009), Gal9 expression on CD34+/CD38- LSCs (*p* = 0.016), the PLT count (*p* = 0.025) and the percentage of PB blasts (*p* = 0.013). IPT lost its significance (Table 8).

**Table 8:** Multivariate analysis for independent factors significantly affecting overall survival in 80 acute lymphoblastic leukemia patients

** Parameter**	*P*-value	**HR**	**95.0% CI for HR**	
			**Lower**	**Upper**
Gender	0.009	3.382	1.348	8.483
Gal-9 on CD34+/CD38-	0.016	3.250	1.245	8.485
PLT group	0.025	4.238	1.199	14.981
PB blasts %	0.013	3.402	1.295	8.936

*PLT* Platelets, *PB* Peripheral Blood, *HR* Hazard ratio, *CI* Confidence interval

### Low Gal9 expression on LSCs and male sex were independent parameters for better PFS

**Fig. 2:**
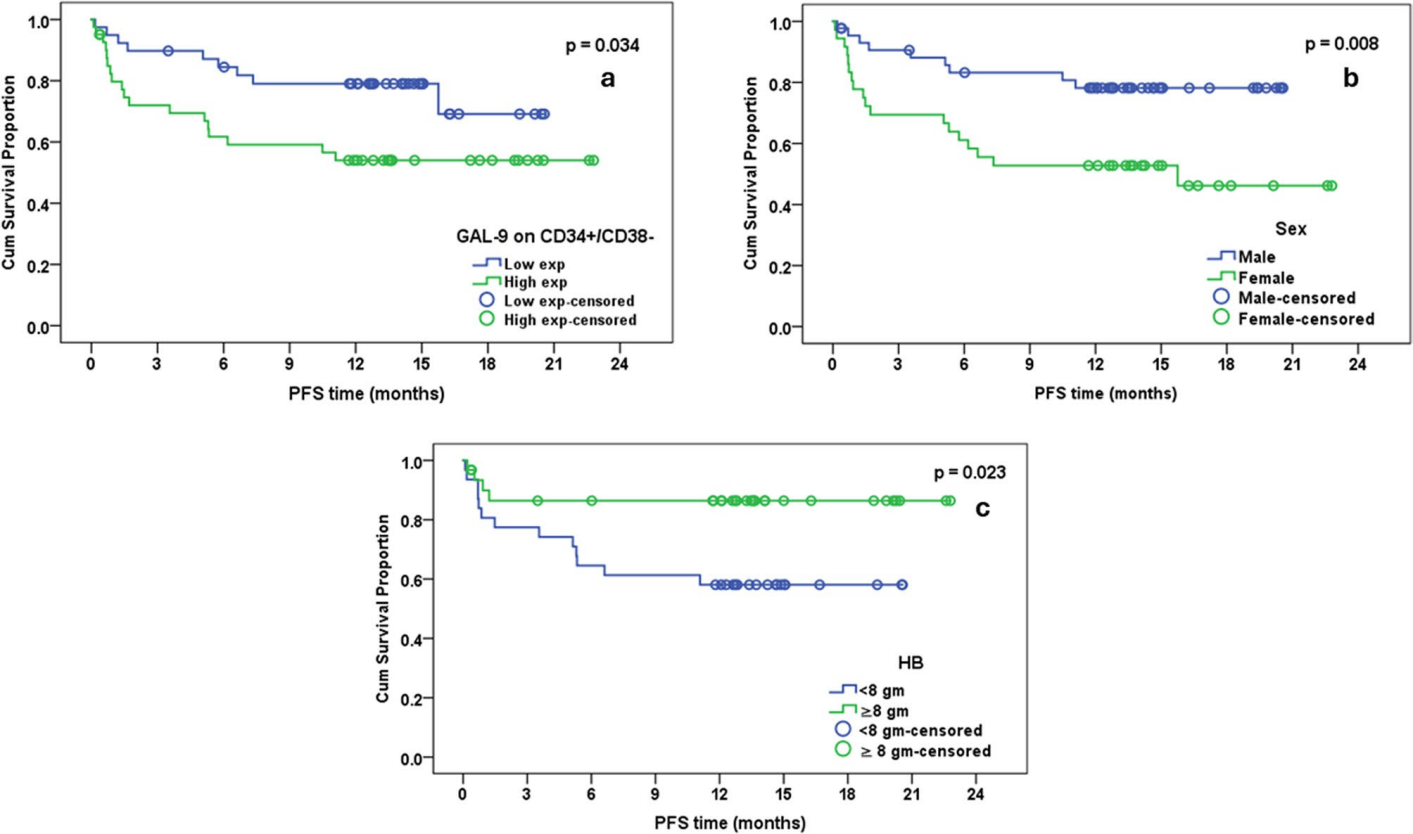
Factors that had significant impact on progression free survival in 80 acute lymphoblastic leukemia patients (using Kaplan-Meier method and log-rank test): (**a**) Gal9 expression on CD34+/CD38- LSCs, (**b**) Sex (44 male and 36 female), (**c**) hemoglobin level in children (cutoff 8 g/dl)

Patients with low Gal9 expression on LSCs had cumulative PFS at 18 months of 69.1% vs. 54.0% for patients with high Gal9 expression **(***P* = 0.034, Fig. 2a). The significance was maintained in multivariate analysis (HR: 2.449, 95% CI: 1.095–5.474, *p* = 0.029).

None of the other ICP markers had an impact on PFS.

Male patients had cumulative PFS of 78.2% at 18 months, whereas female patients had a cumulative PFS of 52.8% (*P* = 0.008, Fig. 2b). The significance was maintained in multivariate analysis; females had a 2.768 h (95% CI; 2.768–6.273; *p* = 0.015).

Pediatric patients with Hb < 8 gm/dl had a PFS of 58.1% at 18 months vs. 86.4% for patients with Hb ≥ 8 gm/dl (*P* = 0.023, Fig. 2c). The significance was lost in multivariate analysis.

All other factors, namely platelet count, TLC, PB and BM blast %, BM cellularity and lymphocyte%, IPT, lymphadenopathy, hepatomegaly, splenomegaly, molecular genetics, cytogenetic risk stratification, CD34+, CD34+/CD38-, CD4+/CD3+, CD8+/CD3+, CD4/CD8 ratio and MRD had no impact on PFS.

## Discussion

In the present study we investigated the expression of two ICP inhibitors, PD1 and TIM3 on BM T lymphocytes and their ligands PDL1 and Gal9 on blast cells and LSCs in 85 newly diagnosed ALL patients.

PD1 expression on CD4 + and CD8 + T-lymphocytes was significantly associated with male sex (*P* = 0.023). In contrast, no association with sex was previously reported [20]. The association with sex is difficult to explain and likely has no clinical significance with the current therapeutic protocols.

No associations were found between PD1 expression on either CD4 + or CD8 + T-.

lymphocytes and age, hepatomegaly, splenomegaly, lymphadenopathy, initial TLC, platelet count, PB or BM blast %, or BM cellularity or lymphocytes %. These findings are consistent with Ruan et al. [20] who studied the expression of PDL1 in both pediatric and adult ALL.

High expression of PD1 on CD4 + T lymphocytes was significantly more common in pediatric patients with Hb levels < 8 gm/dl (*P* = 0.034). This is an association of a good prognostic parameter with a bad one. Such an association was not observed in adults with Hb levels < 10 or ≥ 10 gm/dl. Additionally, no association with Hb was found for its expression on CD8 + T lymphocytes. This finding is consistent with Ruan et al. [20]. We found no significant association between PD1 expression on CD4 + T-lymphocytes and IPT. whereas PD1 expression on CD8 + T-lymphocytes was significantly higher in CALL phenotype (*P* = 0.041); this is also an association of a good prognostic parameter with a bad one. No significant associations were encountered between PD1 expression on CD4 + or CD8 + T-lymphocytes and CD34%, CD34+/CD38-%, CD4+/CD3+, or CD8+/CD3 + T-cell percentages. PD1 expression on CD4 + and CD8 + T-lymphocytes was negatively associated with the CD4:CD8 ratio [(*r*=−0.227, *P* = 0.037) and (*r*=−0.233, *P* = 0.032) respectively]. These findings indicate the coexistence of two good prognostic parameters associated with a better immune status, namely low PD1 expression and a higher CD4/CD8 ratio and vice versa. Additionally, there was no association between PD1 expression on CD4 + or CD8 + T-lymphocytes and molecular genetic translocations, genetic risk groups or the DNA index. These findings are consistent with Ruan et al. [20]

Our results showed higher expression of PD1 on CD4 + and CD8 + T-lymphocytes in the high-risk group than the intermediate and favorable-risk groups; this finding represents an association of two poor prognostic parameters. A similar trend was reported by Kang et al. [21].

In the present study, higher expression was detected in patients with MRD 0.1 to < 10% on day 15, and the difference in expression was significant for CD8+ (*P* = 0.042) and borderline significant for CD4 + T lymphocytes (*P* = 0.051). No association was observed with MRD on day 42. This finding might indicate that higher expression of PD1 on T cells is associated with slower clearance of the malignant clone. No significant association with the achievement of CR was detected. In contrast, Ruan et al. [20] reported no association between the percentages of PD1 + CD4 + or CD8 + T lymphocytes and MRD.

In our study, PD1 expression on either CD4 + or CD8 + T cells had no significant influence on OS or PFS. This finding is in agreement with previous reports [2, 21]. However. Ruan et al. [20] reported that ALL patients with low PD1 expression on CD8 + T lymphocytes have a significant OS advantage over those with high PD1 expression (*P* = 0.028). Additionally, Webster et al. [22] reported that the addition of PD1 and CTLA-4 blockade to Blinatumomab was significantly associated with improved CR rate, MRD negativity and OS in R/R adult ALL.

This discrepancy between our results and those of other studies might be attributed to differences in the studied cohorts, small sample sizes or different follow-up periods. Away from statistical significance, it is advisable to evaluate PD1 and other ICP inhibitors at the individual case level for potential targeted therapy.

In the present study, we found no associations between TIM3 expression on CD4 + or CD8 + T-lymphocytes and sex, lymphadenopathy, hepatomegaly, or splenomegaly. This is consistent with Hohtari et al. [23].

There was no association between TIM3 expression on CD4 + T-lymphocytes and TLC, platelet count, PB blast percentage or Hb levels in children or adults. Additionally, there was no association between TIM3 expression on CD8 + T-lymphocytes and platelets or Hb level in children or adults, BM cellularity, BM lymphocyte percentages, IPT, CD34%, CD34+/CD38-%, CD4+/CD3+, CD8+/CD3 + T-cell percentages, or the CD4/CD8 ratio. However, high expression of TIM3 on CD8 + T lymphocytes was significantly more common in patients with a TLC < 10 × 10^9^/L (*P* = 0.026). A significantly higher median initial PB blast percentage (*P* = 0.007) was found in patients with lower TIM3 expression on CD8 + T lymphocytes.

A significantly higher median initial BM blast percentage (*P* = 0.030) was found in patients with lower TIM3 expression on CD4 + T lymphocytes, whereas a borderline significant association was observed for expression on CD8 + T-lymphocytes (*P* = 0.051). In contrast, Hohtari et al. [23] reported an association of TIM3 expression on CD4 + T lymphocytes and a high BM blast percentage (*P* = 0.003), but no other associations with clinical parameters were found.

In the present study, TIM3 expression on CD4 + and CD8 + was negatively correlated with TLC, and BM blasts. TIM3 expression on CD8 + was also negatively correlated with the percentage of PB blasts. This represents prognostic parameters that act in the same direction.

No statistically significant association was found between TIM3 and day 15 MRD. Day 42 MRD was associated with higher TIM3 expression on CD8 + T lymphocytes; high TIM3, a bad prognostic parameter, is expected to be associated with positive MRD. Paradoxically, CR at the end of induction was associated with high TIM3 expression (*P* = 0.011), which is difficult to explain. However, any single prognostic parameter should be viewed in the context of all other factors and should be tested in a multivariate platform to verify its true impact.

In our study, TIM3 expression on either CD4 + or CD8 + T cells had no significant impact on OS or PFS. Our results are consistent with Akbar et al. [24]. In contrast, Blaeschke et al. [2] reported high TIM3 expression on BM T-cells in relapsing patients. This might be explained by the long 6 year follow up which allowed the detection of relapsed patients.

In the present study, there was no association between PD1/TIM3 co-expression on CD4 + or CD8 + T-lymphocytes and age, sex, lymphadenopathy, hepatomegaly or splenomegaly. Hohtari et al. [23] also, reported no significant relationship between PD1/TIM3 co-expression on CD4 + or CD8 + T-lymphocytes and patients’ age. Other factors have not been previously addressed.

No associations were detected between PD1/TIM3 co-expression on T lymphocytes and Hb level, platelet count, BM cellularity or BM lymphocytes, CD34+, CD34+/CD38-, CD3+/CD4+, CD3+/CD8+, the CD4/CD8 ratio, molecular genetics, cytogenetic risk groups or the DNA index. This was not previously addressed.

A TLC < 10 × 10^9^/L was associated with high PD1/TIM3 co-expression on CD8 + T cells (*p* = 0.005). A similar trend was observed for co-expression on CD4 + T cells (*p* = 0.085).

A positive association was detected between high PD1/TIM3 co-expression on CD8 + but not CD4 + T cells and CALL and pre-B vs. pro-B phenotype (*p* = 0.016). PB blast percentage was associated with low co-expression on CD8 + but not CD4 + T cells (*P* = 0.004). BM blast percentage was significantly associated with low co-expression on CD4 + but not CD8 + T cells (*p* = 0.04). These associations were not previously addressed. The associations between good and poor prognostic parameters are paradoxical. These findings indicate that each parameter affects prognosis through a different mechanism. In biological systems, the sum of factors, rather than any single factor, determines the prognosis.

There was no association between PD1/TIM3 co-expression on either CD4 + or CD8 + T-lymphocytes and MRD or CR at the end of induction. Moreover, there was no impact on OS or PFS. This is partially in contrast to Hohtari et al. [23] and Blaeschke et al. [2] who reported that PD1/TIM3 co-expression was associated with a significantly reduced probability of RFS but not OS. Hohtari et al. [23] reported that high proportion of PD1+/TIM3 + double positive T cells, older age, and low platelet count at diagnosis were associated with poor survival.

In our study, there was no association between PDL1 expression on blasts or CD34+/CD38- LSCs and age, sex, lymphadenopathy, or splenomegaly. In contrast, higher expression in adults was previously reported [25]. Our results revealed that significantly higher PDL1 expression on CD34+/CD38- LSCs but not on blasts was associated with hepatomegaly (*P* = 0.028). This finding indicates that higher expression is associated with extramedullary involvement; both are bad prognostic parameters.

We found no significant associations between PDL1 expression on blasts or CD34+/CD38- LSCs and TLC, platelet count, PB blast%, Hb level in children or adults, BM cellularity, IPT, BM blasts or lymphocytes%. Hamdan et al. [25] also, reported that PDL1 expression was not associated with blast count or IPT.

In our study, a significantly higher median CD4+/CD3+% (*P* = 0.014) and CD8+/CD3+% (*P* = 0.030) were found in patients with low PDL1 expression on blasts. These findings indicate that a greater number of immune cells is associated with lower expression of PDL1 on blast cells. Additionally, a significantly greater median CD34% (*P* = 0.004) was found in patients with high PDL1 expression on blasts. In these patients, parameters of similar good prognostic impact are hand in hand. On the other hand, no significant associations were detected with IPT, the CD34+/CD38- percentage, or the CD4/CD8 ratio.

In the present study, PDL1 expression on blasts was positively correlated with the percentage of CD34 + cells and the percentage of CD34+/CD38- LSC cells and negatively correlated with the percentage of CD4+/CD3 + T cells and the percentage of CD8+/CD3 + T cells. These are correlations of parameters with similar prognostic significance.

PDL1 expression on CD34+/CD38- LSCs was negatively correlated with the percentage of CD34 + cells and the percentage of CD34+/CD38 + cells. This finding indicates that the biological characteristics of LSCs, not just their number, might be important.

In our study, PDL1 expression on CD34+/CD38- LSCs was significantly lower in t(12:21) positive patients (*P* = 0.049). This is another situation in which two good prognostic parameters are hand in hand. However, we found no statistically significant associations with other molecular genetic findings, genetic risk groups or the DNA index. Hamdan et al. [25] reported that PDL1 expression was not associated with cytogenetic findings. Kang et al. [21] reported a trend toward increased median PDL1 expression on blast cells.

In our study, significantly increased PDL1 expression on blasts, but not on CD34+/CD38- LSCs, was more frequently detected in patients with day 15 MRD 0.1-<10% (*P* = 0.047) than in patients with day 15 MRD < 0.1. No associations were found with day 42 MRD, or with CR at the end of induction. In this case two poor prognostic parameters are hand in hand.

In our study, PDL1 expression on blasts or CD34+/CD38- LSCs had no significant impact on patient’s OS or PFS. These findings are consistent with previous studies (2, 25); but in contrast with Kang et al. [21], who reported that RFS at 4 years was significantly greater in patients with low PDL1 expression on blasts (*P* = 0.049). The difference between the latter study and other studies, including ours, may be attributed to the long follow up period allowing for relapse to occur in some patients.

In the present study there was no association between Gal9 expression on blasts or CD34+/CD38- LSCs and age, sex, lymphadenopathy, or splenomegaly. A significantly higher Gal9 expression on LSCs, but not on blasts, was associated with hepatomegaly (*P* = 0.015). This is another example of an association of two poor prognostic parameters; high expression of Gal9 on LSCs and extramedullary disease.

In the present study, there was no association between Gal9 expression on blasts and TLC, platelet count, PB blast percentage, Hb level in children or adults, BM cellularity, BM blasts or lymphocyte percentages. A significantly greater median initial PB blast percentage (*P* = 0.020) and BM blasts percentage (*P* = 0.030) were found in patients with low Gal9 expression, whereas a significantly greater median initial BM lymphocyte percentage (*P* = 0.034) was found in patients with high Gal9 expression. An association of contradictory prognostic parameters; however, the high lymphocytes may include T-regs, not just the effector T- cells.

No significant associations were detected between Gal9 expression on blasts and IPT, CD34%, CD34+/CD38-%, CD4+/CD3 + or CD8+/CD3+ % T-cells percentage or the CD4/CD8 ratio. No associations were detected between Gal9 expression on LSCs and IPT, CD4+/CD3+, CD8+/CD3+ % T-cells, or the CD4/CD8 ratio. A significantly greater median CD34% (*P* = 0.002) and CD34+/CD38-% (*P* = 0.002) were found in patients with low Gal9 expression on CD34+/CD38- LSCs. Thus, both the number and the biological characteristics of LSCs should be taken into consideration when evaluating the impact of LSCs on disease outcome. Gal9 expression on LSCs showed negative correlations with CD34+ % (*r*=−0.394, *P* < 0.001) and CD34+/CD38- % (*r*=−0.414, *P* < 0.001). This further emphasizes that the characteristics of the LSCs might be equally important to the number. In other words, the nature, not only the number, of LSCs is the cause of resistance to therapy.

In our study, a significantly higher Gal9 expression on blasts was detected in t(9;22) p210 positive patients (*P* = 0.045). Another example of an association between two poor prognostic parameters. On the other hand, there were no statistically significant associations with other molecular genetic findings, genetic risk groups or DNA indices.

In our study, patients with lower Gal9 expression on CD34+/CD38- LSCs had significantly longer OS and PFS than patients with high Gal9 expression (*P* = 0.026 and *P* = 0.034 respectively). This finding is in line with the importance of LSC characteristics as prognostic factors. On the other hand, Gal9 expression on blasts had no significant influence on OS or PFS. This finding is in concordance with a previous study by Akbar et al. [24] who also denied an impact on relapse.

In the present study, no associations were detected between PDL1/Gal9 co-expression on blasts and age, sex, lymphadenopathy, hepatomegaly, splenomegaly, TLC, platelet count, PB blast percentage, Hb level in children or adults, BM cellularity, BM blast or lymphocyte percentages, IPT, CD34%, CD34+/CD38-%, CD4+/CD3 + or CD8+/CD3+% T-cell or the CD4/CD8 ratio. PDL1/Gal9 co-expression on blasts was positively correlated with the percentage of CD34 + cells and the percentage of CD34+/CD38- cells and negatively correlated with the percentage of CD4+/CD3 + cells, i.e., positively correlated with the primitive cells driving the malignant condition and negatively correlated with the parameter suppressing it.

A significantly higher PDL1/Gal9 co-expression on blasts was detected in t(9;22) p210 positive patients (*P* = 0.029). Another situation involving the association of two poor prognostic parameters. On the other hand, no statistically significant associations were found with other molecular genetic findings, genetic risk groups, the DNA index, CD34%, CD34+/CD38-% LSCs, MRD, CR at the end of induction, OS or PFS.

However, PDL1/Gal9 co-expression on CD34+/CD38-% LSCs was negatively associated with CD34% (*p* = 0.003) and CD34+/CD38-% (*p* = 0.002). As already mentioned, this reflects the importance of both the number and biological characteristics of LSCs. 

To the best of our knowledge, the combination of PDL1 and Gal9 co-expression in ALL has not been previously reported. Co-evaluations of various parameters are important for revealing the various contributions of each parameter and the potential synergistic effects of their co-expression.

In the multivariate analysis, the independent factors that significantly affected OS were male sex and Gal9 expression on CD34+/CD38- LSCs with the PLT count and the initial PB blasts percentage showing borderline significance. The independent factors that significantly affected PFS were male sex and Gal9 expression on CD34+/CD38-LSCs. Thus, Gal9 expression on LSCs is an independent prognostic parameter for both OS and PFS. This finding has not been previously reported. Previous independent factors affecting PFS included age, TLC, MRD and TIM3 expression on CD4 + BM T cells [2].

The results of ICI markers expression in our study revealed that the higher expression of these markers is associated with poor prognostic parameters in some instances and with good prognostic parameters in others. These paradoxical findings indicate that different prognostic parameters may have different mechanisms of action. Which parameters to be tested depends on the aim. For the sake of a standard therapeutic approach, we opt for classical parameters. For the sake of targeted therapy, specific parameters should be sought.

Our results suggest that acute leukemia BM has a unique immune cell composition that is significantly associated with several prognostic hematological and clinical parameters and the response to therapy. The inconsistency between our results and some of the results reported in the literature, regarding disease outcome, may be attributed in some cases to different treatment protocols and longer follow up periods in other studies.

A limitation of this study is that the reported marker expressions are quantitative findings and are merely descriptive by nature. Further functional studies are needed to elucidate their effects on the switch from immune activation to an immunosuppressive state. Additionally, further research clarifying the impact of acute leukemia immunobiology on disease progression and how to translate immunophenotype features into biomarkers for use in treatment is imperative.

Another limitation is the relatively small number of patients in some subgroups and the short follow up period. Therefore, further studies are needed to expand on our current findings and explore the therapeutic potential of ICI inhibitors in acute lymphoblastic leukemia.

## Supplementary Information

Supplementary Material 1

Supplementary Material 2

Supplementary Material 3

Supplementary Material 4

Supplementary Material 5

Supplementary Material 6

Supplementary Material 7

## Data Availability

All data generated or analysed during this study are included in this article [and its supplementary information files].

## Abbreviations

BCP-ALL: B-precursor ALL
ICI: Immune check point inhibitors
CTLA-4: Cytotoxic T lymphocyte antigen 4
PD-1: Programmed cell death-1
TIM3: T-cell immunoglobulin and mucin domain 3
PDL-1: Programmed cell death-1 ligand
Lag-9: Lymphocyte activation gene-9
LSCs: leukemia stem cells
Mo Ab: Monoclonal antibodies
