# Supplementary material for: PD1/PDL1 and TIM3/Gal9 expression in acute lymphoblastic leukemia: Gal-9 expression on leukemia stem cells as an independent prognostic parameter
Source: BMC Cancer. 2025 Sep 12;25:1421. doi: 10.1186/s12885-025-14856-9 (PMC12432999; doi:10.1186/s12885-025-14856-9)
Supplement: Supplementary file 1 — Supplementary Material 1 [file 12885_2025_14856_MOESM1_ESM.docx]

**Supplementary table (1): List of Monoclonal antibodies for immune check point inhibitors**

| **Monoclonal**  **Antibody** | **Fluorochrome** | **Clone** | **Isotype** | **Source** | **Cat. No.** |
| --- | --- | --- | --- | --- | --- |
| **CD 279**  **(PD-1)** | PE-Cy7 | EH12.1 | Mouse IgG1, Kappa | BD Pharmingen**^TM^** | 561272 |
| **CD274 (PDL-1)** | APC-R700 | MIH1 | Mouse IgG1, Kappa | BD Horizon**^TM^** | 565188 |
| **CD366 (TIM-3)** | Alexa Fluor 647 | 7D3 | Mouse IgG1, Kappa | BD Pharmingen**^TM^** | 565558 |
| **Galectin-9** | Alexa Fluor 488 | 1015214 | Mouse IgG2A | R&D Systems | FAB20453G |
