# Supplementary material for: PD1/PDL1 and TIM3/Gal9 expression in acute lymphoblastic leukemia: Gal-9 expression on leukemia stem cells as an independent prognostic parameter
Source: BMC Cancer. 2025 Sep 12;25:1421. doi: 10.1186/s12885-025-14856-9 (PMC12432999; doi:10.1186/s12885-025-14856-9)
Supplement: Supplementary file 2 — Supplementary Material 2 [file 12885_2025_14856_MOESM2_ESM.docx]

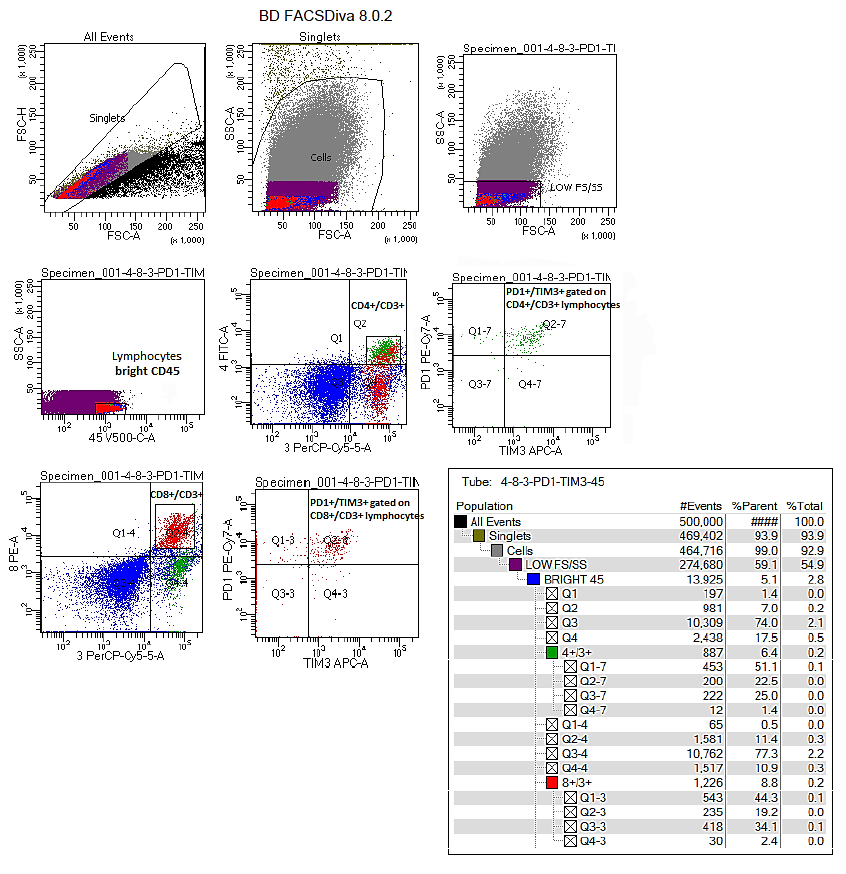


**Supplementary Figure (1): PD1 and TIM3 expression on CD4+/CD3+ and CD8+/CD3+ T lymphocytes:** A primary gate was constructed on CD45 bright population (lymphocytes) then secondary gates were taken on CD3+/CD4+ and CD3+/CD8+ populations for detection of PD-1and TIM3 expression on CD4+ and CD8+ T lymphocytes respectively.
