# Supplementary material for: PD1/PDL1 and TIM3/Gal9 expression in acute lymphoblastic leukemia: Gal-9 expression on leukemia stem cells as an independent prognostic parameter
Source: BMC Cancer. 2025 Sep 12;25:1421. doi: 10.1186/s12885-025-14856-9 (PMC12432999; doi:10.1186/s12885-025-14856-9)
Supplement: Supplementary file 3 — Supplementary Material 3 [file 12885_2025_14856_MOESM3_ESM.docx]

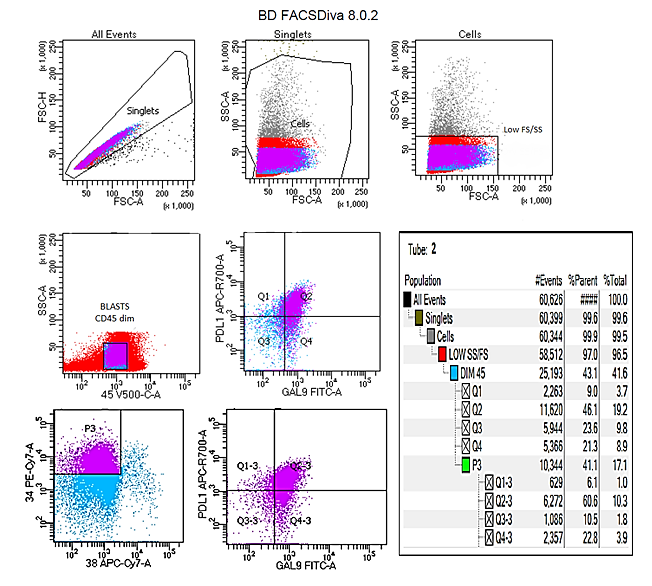


**Supplementary Figure (1): PDL-1 and Gal-9 expression on blast cells and LSCs**.

A primary gate was constructed on CD45 dim population and expression of PDL-1 and Gal9 on blast cells (dim for CD45) was detected. A secondary gating on CD34+/CD38- was done for detection of PDL1 and Gal9 expression on LSCs.
