# Supplementary material for: PD1/PDL1 and TIM3/Gal9 expression in acute lymphoblastic leukemia: Gal-9 expression on leukemia stem cells as an independent prognostic parameter
Source: BMC Cancer. 2025 Sep 12;25:1421. doi: 10.1186/s12885-025-14856-9 (PMC12432999; doi:10.1186/s12885-025-14856-9)
Supplement: Supplementary file 4 — Supplementary Material 4 [file 12885_2025_14856_MOESM4_ESM.docx]

**
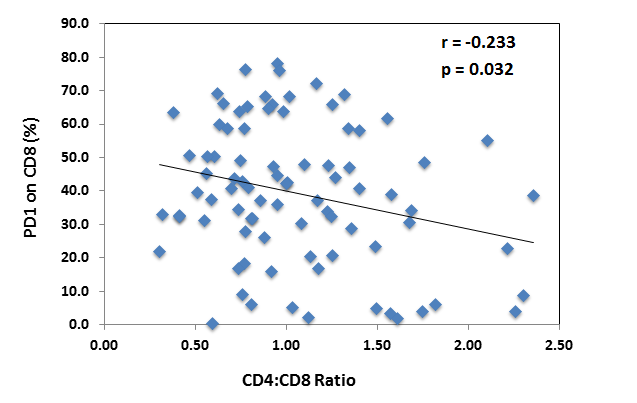

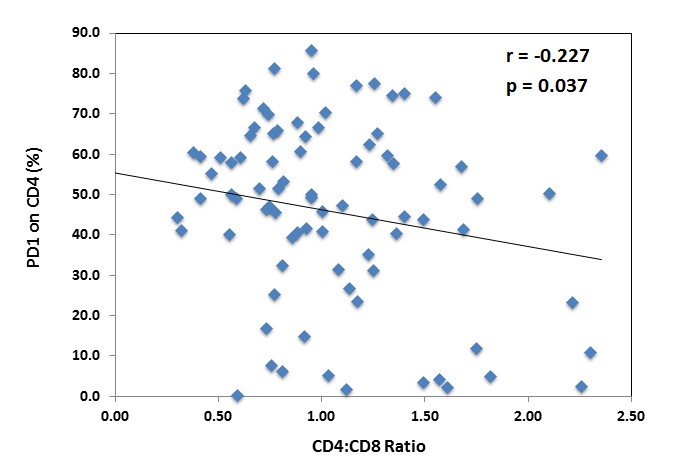
**

**b**

**a**

**
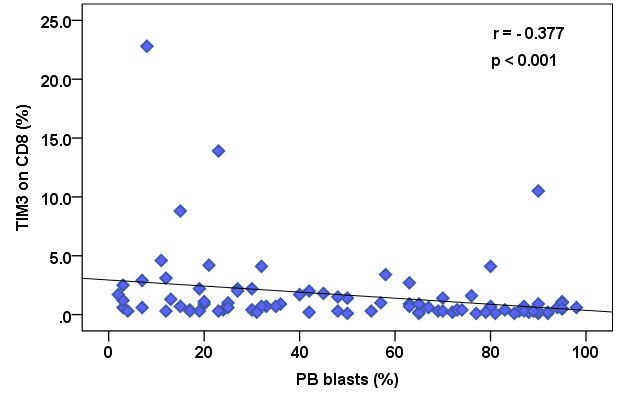

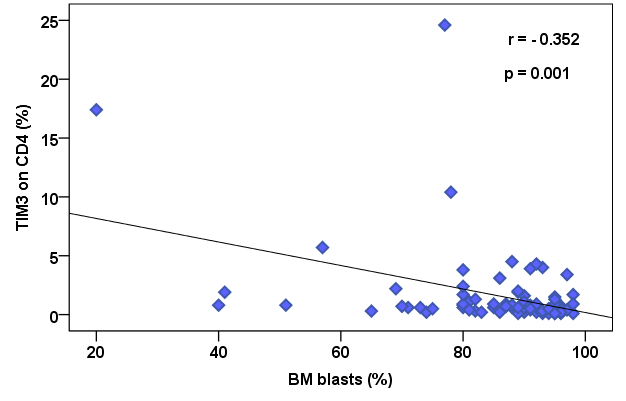
**

**d**

**c**

**
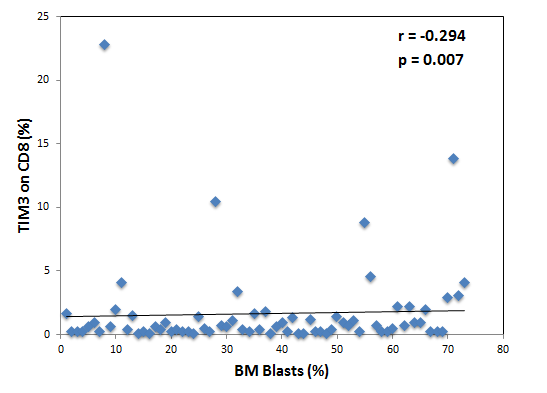
**


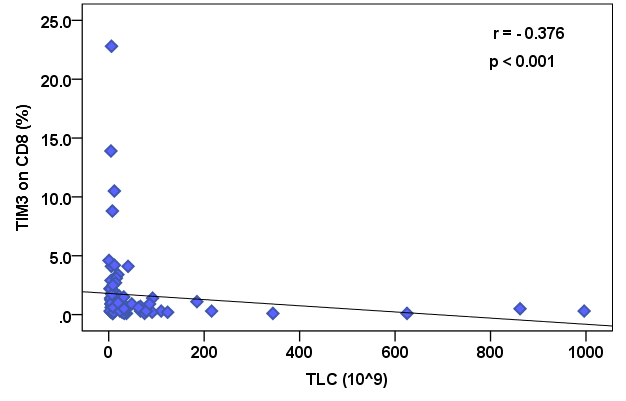


**f**

**e**

**
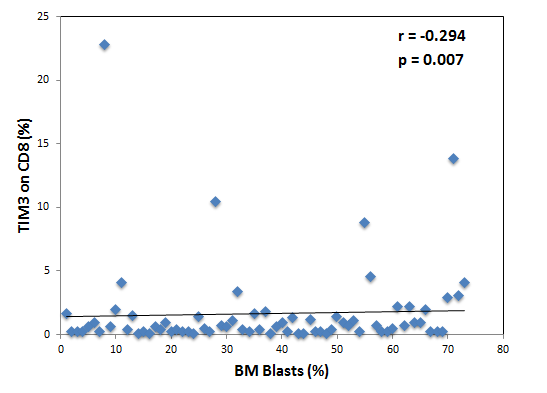
**

**g**

**Supplementary Fig (3)**

**Significant correlations of PD1 expression on T lymphocytes with parameters in 85 ALL patients**

1. expression on CD4+ T lymphocytes (%) with the CD4:CD8 ratio
2. expression on CD8+ T lymphocytes (%) with the CD4:CD8 ratio
3. expression on CD4+ T lymphocytes (%) with TLC

**Significant correlations of TIM-3 expression on T lymphocytes with parameters in 85 ALL patients**

1. expression on CD4+ T lymphocytes (%) with BM blasts (%)
2. expression on CD8+ T lymphocytes (%) with PB blasts (%)
3. expression on CD8+ T lymphocytes (%) with TLC (10^9/L)
4. expression on CD8+ T lymphocytes (%) with BM blasts
