# Supplementary material for: PD1/PDL1 and TIM3/Gal9 expression in acute lymphoblastic leukemia: Gal-9 expression on leukemia stem cells as an independent prognostic parameter
Source: BMC Cancer. 2025 Sep 12;25:1421. doi: 10.1186/s12885-025-14856-9 (PMC12432999; doi:10.1186/s12885-025-14856-9)
Supplement: Supplementary file 5 — Supplementary Material 5 [file 12885_2025_14856_MOESM5_ESM.docx]

**
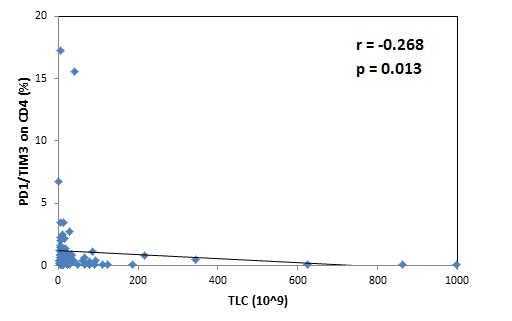

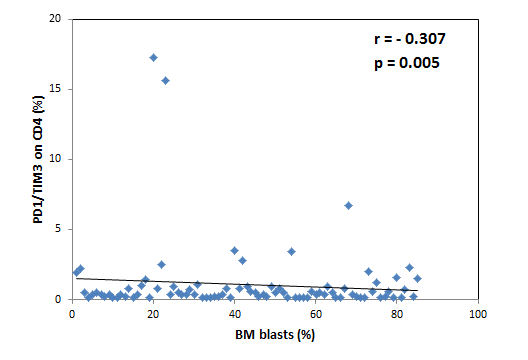
**

**a**b

a

**b**b

**a**

**b**

**a**

**
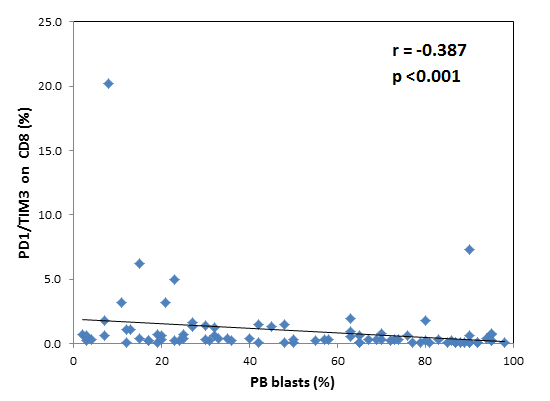
**

**
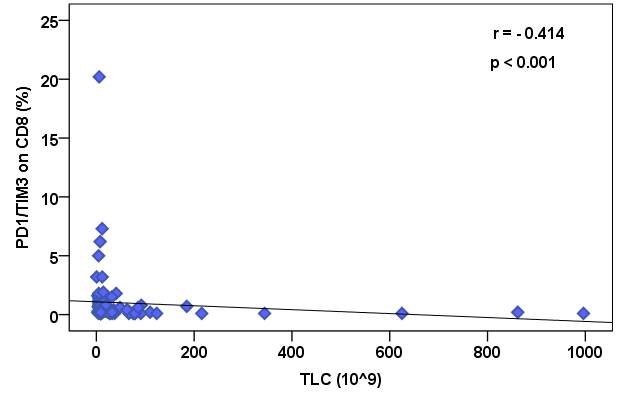
**

**c**

**d**

**c**

**a**

**Supplementary Fig (4) Correlations of PD1 /TIM3 co-expression with parameters in 85 ALL patients:**

1. Co-expression of PD-1 /TIM-3 on CD4+ T lymphocytes with TLC
2. Co-expression of PD-1 /TIM-3 on CD4+ T lymphocytes with BM blast %
3. Co-expression of PD-1 /TIM-3 on CD8+ T lymphocytes with TLC
4. Co-expression of PD-1 /TIM-3 on CD8+ T lymphocytes with PB blast%
