# Supplementary material for: PD1/PDL1 and TIM3/Gal9 expression in acute lymphoblastic leukemia: Gal-9 expression on leukemia stem cells as an independent prognostic parameter
Source: BMC Cancer. 2025 Sep 12;25:1421. doi: 10.1186/s12885-025-14856-9 (PMC12432999; doi:10.1186/s12885-025-14856-9)
Supplement: Supplementary file 6 — Supplementary Material 6 [file 12885_2025_14856_MOESM6_ESM.docx]

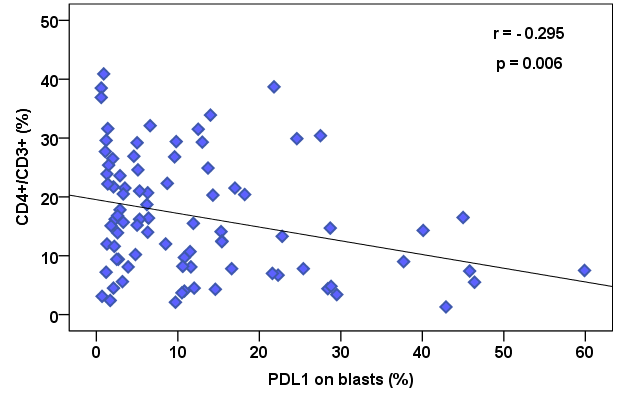

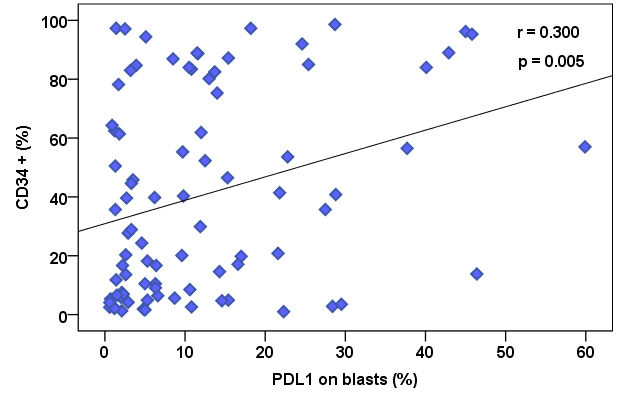

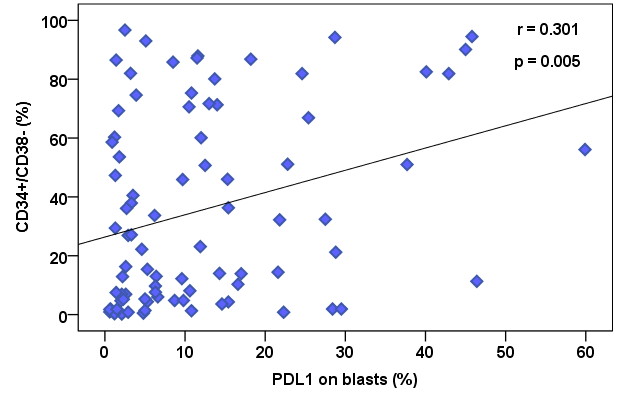


**c**

**b**

**a**


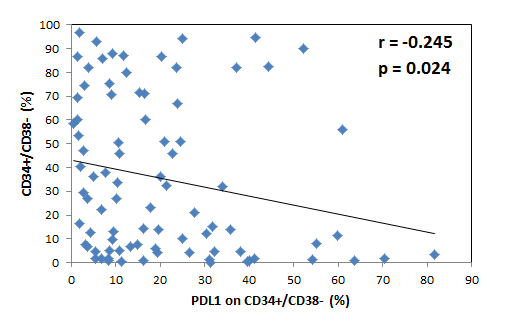

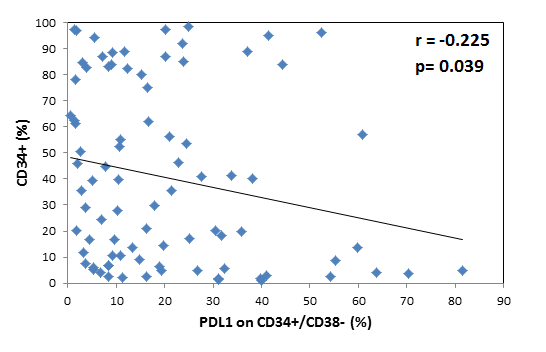

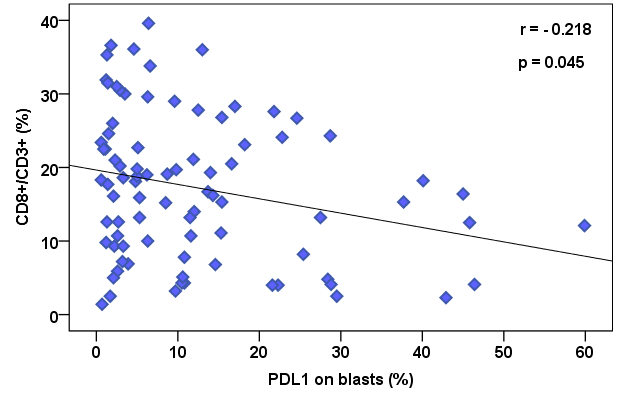

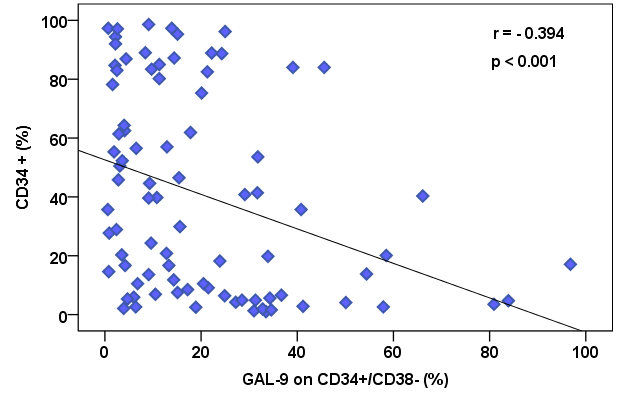

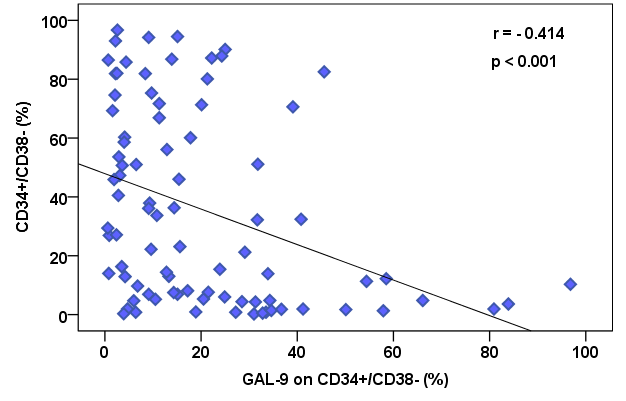


**e**

**d**

**f**

**g**

**h**

**Supplementary Figure (5) Correlations of PDL1 and Gal9 with parameters in 85 ALL cases**

1. Correlation of CD34+ (%) with PDL1 expression on blasts (%).
2. Correlation of PDL1 on blasts % with CD34+/CD38- (%)
3. Correlation of PDL1 on blasts % with CD4+/CD3+ %
4. Correlation of PDL1 on blasts % with CD8+/CD3+ %
5. Correlation of PDL1 on CD34+/CD38-% with CD34 %
6. Correlation of PDL1 on CD34+/CD38-% with CD34+/CD38- %
7. Correlation of Gal9 expression on CD34+/CD38-% with CD34 %
8. Correlation of Gal9 expression on CD34+/CD38-% with CD34+/CD38-%
