# Supplementary material for: PD1/PDL1 and TIM3/Gal9 expression in acute lymphoblastic leukemia: Gal-9 expression on leukemia stem cells as an independent prognostic parameter
Source: BMC Cancer. 2025 Sep 12;25:1421. doi: 10.1186/s12885-025-14856-9 (PMC12432999; doi:10.1186/s12885-025-14856-9)
Supplement: Supplementary file 7 — Supplementary Material 7 [file 12885_2025_14856_MOESM7_ESM.docx]

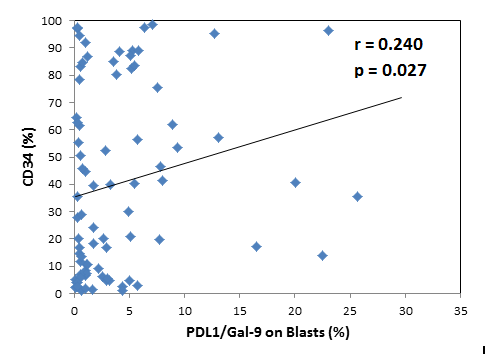

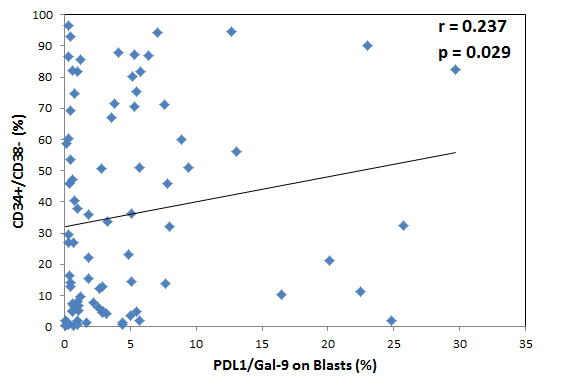


**b**

**a**


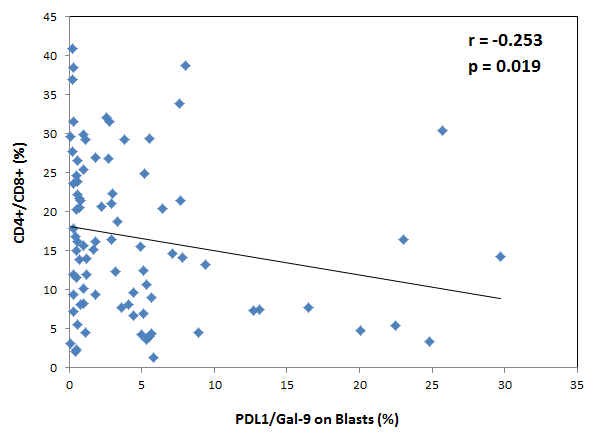

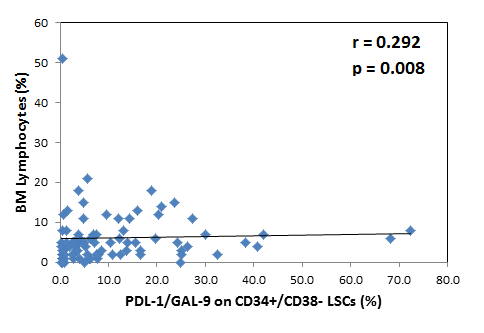


**c**

**d**


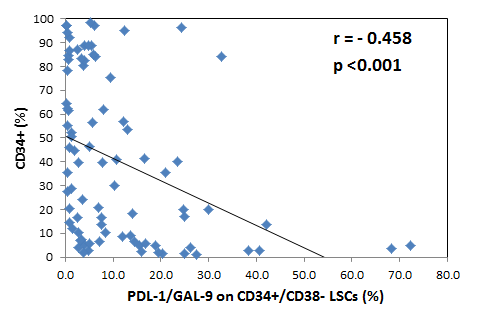

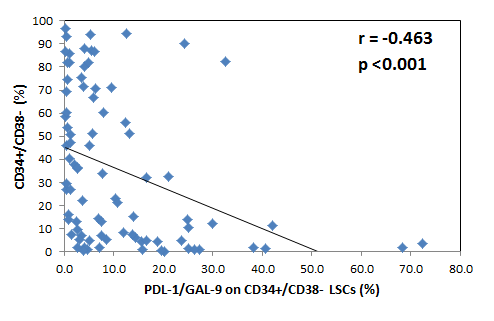


**e**

**f**

**Supplementary Figure (6)**

**a: Correlation of PDL1/Gal9 on blasts with CD34%**

**b: Correlation of PDL1/Gal9 on blasts with CD34+/CD38-%**

**c: Correlation of PDL1/Gal9 on blasts with CD4:CD8 ratio**

**d: Correlation of PDL1/Gal9 on CD34+/CD38- LSCs with BM lymphocyte %**

**e: Correlation of PDL1/Gal9 on CD34+/CD38- LSCs with CD34+ %**

**f: Correlation of PDL1/Gal9 on CD34+/CD38- LSCs with Cd34+/CD38- %**
